# Establishment of a Mouse Model with Misregulated Chromosome Condensation due to Defective *Mcph1* Function

**DOI:** 10.1371/journal.pone.0009242

**Published:** 2010-02-16

**Authors:** Marc Trimborn, Mahdi Ghani, Diego J. Walther, Monika Dopatka, Véronique Dutrannoy, Andreas Busche, Franziska Meyer, Stefanie Nowak, Jean Nowak, Claus Zabel, Joachim Klose, Veronica Esquitino, Masoud Garshasbi, Andreas W. Kuss, Hans-Hilger Ropers, Susanne Mueller, Charlotte Poehlmann, Ioannis Gavvovidis, Detlev Schindler, Karl Sperling, Heidemarie Neitzel

**Affiliations:** 1 Institute for Medical Genetics, Charité – Universitätsmedizin Berlin, Berlin, Germany; 2 Institute of Human Genetics, Charité – Universitätsmedizin Berlin, Berlin, Germany; 3 Max Planck Institute for Molecular Genetics, Berlin, Germany; 4 Center for Stroke Research Berlin, Charité – Universitätsmedizin Berlin, Berlin, Germany; 5 Institute of Human Genetics, University Wuerzburg, Wuerzburg, Germany; Brunel University, United Kingdom

## Abstract

Mutations in the human gene *MCPH1* cause primary microcephaly associated with a unique cellular phenotype with premature chromosome condensation (PCC) in early G2 phase and delayed decondensation post-mitosis (PCC syndrome). The gene encodes the BRCT-domain containing protein microcephalin/BRIT1. Apart from its role in the regulation of chromosome condensation, the protein is involved in the cellular response to DNA damage. We report here on the first mouse model of impaired *Mcph1*-function. The model was established based on an embryonic stem cell line from BayGenomics (RR0608) containing a gene trap in intron 12 of the *Mcph1* gene deleting the C-terminal BRCT-domain of the protein. Although residual wild type allele can be detected by quantitative real-time PCR cell cultures generated from mouse tissues bearing the homozygous gene trap mutation display the cellular phenotype of misregulated chromosome condensation that is characteristic for the human disorder, confirming defective *Mcph1* function due to the gene trap mutation. While surprisingly the DNA damage response (formation of repair foci, chromosomal breakage, and G2/M checkpoint function after irradiation) appears to be largely normal in cell cultures derived from Mcph1^gt/gt^ mice, the overall survival rates of the Mcph1^gt/gt^ animals are significantly reduced compared to wild type and heterozygous mice. However, we could not detect clear signs of premature malignant disease development due to the perturbed *Mcph1* function. Moreover, the animals show no obvious physical phenotype and no reduced fertility. Body and brain size are within the range of wild type controls. Gene expression on RNA and protein level did not reveal any specific pattern of differentially regulated genes. To the best of our knowledge this represents the first mammalian transgenic model displaying a defect in mitotic chromosome condensation and is also the first mouse model for impaired *Mcph1-*function.

## Introduction

Mitosis is the fundamental process ensuring the proper segregation of replicated chromatids to the daughter cells. At the beginning of mitosis the apparently amorphous interphase chromatin is organized into individual chromosomes with a pair of separated sister chromatids. This process is referred to as chromosome assembly, implying chromosome condensation and sister chromatid resolution [1], [2]. The assembled chromosomes become attached to the spindle tubules in prometaphase and sister chromatids are segregated into daughter cells in anaphase. Among the earliest events in mitosis are the onset of chromosome condensation and the separation of mature centrosomes. Cell entry into mitosis is under the control of a tightly regulated network of protein kinases, cyclins and protein phosphatases [3]. Understanding the structural and molecular basis of mitotic chromosome condensation and segregation remains a basic challenge in cell biology.

The first description of a disorder in man affecting chromosome condensation was reported in 2002 (OMIM 606858) [4]. Cultures of patients cells display high numbers of prophase-like cells (up to 20%) due to premature chromosome condensation ( =  PCC syndrome) in early G2-phase commencing already 1 hour after completion of S-phase and delayed decondensation post-mitosis. In 2004, we demonstrated that PCC syndrome and MCPH1 primary microcephaly (OMIM 251200) are allelic disorders, both caused by mutations in the *MCPH1* gene (GeneID 79648) encoding microcephalin/BRIT1 [5], [6]. The two conditions share the common cellular phenotype of misregulated chromosome condensation. We demonstrated that siRNA-mediated depletion of *MCPH1*-mRNA is sufficient to reproduce this phenotype and that the condensation defect is mediated by condensin II [5], [7]. These findings implicated microcephalin/BRIT1 as a novel regulator of chromosome condensation and linked the apparently disparate fields of neurogenesis and chromosome biology.

The full length human *MCPH1*-encoded protein microcephalin/BRIT1 encompasses 835 amino acids and has one N-terminal and two C-terminal BRCT-domains (BRCT  =  BRCA1 C-terminal). The mouse ortholog shares the same domain structure and encodes a protein of 822 amino acids. The structure is evolutionary conserved in metazoans. Genes with an equivalent structure are even found in the two main invertebrate model organisms, *Drosophila melanogaster* (GeneID 36372) and *Caenorhabditis elegans* (GeneID 173252) [8].

BRCT-domains are found predominantly in proteins involved in cell cycle checkpoint control and DNA repair [9], [10]. Therefore, it was speculated that microcephalin/BRIT1 might play a role in DNA damage response and checkpoint control, in addition to its role in chromosome condensation. Evidence that microcephalin/BRIT1 might be involved in checkpoint control comes mainly from experiments using RNA interference. A severe impairment of the G2/M DNA damage checkpoint in human U2OS cells was reported after depletion of microcephalin/BRIT1 by RNAi. Microcephalin/BRIT1-depleted U2OS cells displayed a loss of G2/M checkpoint control allowing cells to proceed into mitosis despite exposure to considerable doses of ionizing irradiation (3–10 Gy) [11], [12]. Furthermore, protein levels of the checkpoint mediator BRCA1 and the checkpoint kinase CHK1 were decreased following treatment with *MCPH1*-siRNAs. However, in patient cells with *MCPH1* truncating mutations no downregulation of BRCA1 and CHK1 was observed [13]. Alderton et al. found microcephalin/BRIT1 to function downstream of CHK1 in the ATR-dependent damage response pathway that affects CDC25A stability and G2/M-checkpoint arrest after replication fork stalling.

Rai et al. postulated that *MCPH1* is a tumor suppressor gene. Copy number and expression of *MCPH1* were reduced in 35 of 87 (40%) advanced epithelial ovarian tumors and in 72% of 54 breast cancer specimens. *MCPH1* expression was inversely correlated with genomic instability and metastasis. They also detected a mutation resulting in a premature stop codon in exon 11 and subsequent deletion of the C-terminal BRCT-domains in one of ten breast cancer samples. There was loss of heterozygosity in the other allele of *MCPH1* [14]. Furthermore, these authors showed that microcephalin/BRIT1 co-localizes to DNA damage response proteins, such as MDC1, 53BP1, NBS1, and phosphorylated ATM and is required for the activation of these proteins. The mutated *MCPH1*-transcript detected in the breast cancer specimen described above was unable to complement the defective activation of DNA damage response proteins, in contrast to full length *MCPH1*.

Recently, it was reported that the ability of *hMCPH1* to localize to the sites of DNA double-strand breaks depends on its C-terminal tandem BRCT domains [15] and is mediated by phosphorylated H2AX–termed γH2AX. These findings correlate with data obtained by the cloning and expression of chicken *Mcph1* [16]. Also very recently it was shown that microcephalin/BRIT1 not only interacts with condensin II in the regulation of chromosome condensation but also in the homologous repair of DNA damage [17].

In addition, microcephalin/BRIT1 appears to have also centrosomal functions. Prolonged treatment of *MCPH1* patient lymphoblastoid cell lines with nocodazole results in supernumerary centrosomes, a feature also observed in Seckel syndrome [13]. It was demonstrated in model organisms that isoforms of microcephalin/BRIT1 localize to the centrosome [16], [18]. In *Drosophila* it appears to be essential for the coordination of mitosis in the syncytical embryo as in *mcph1* mutant flies mitotic entry is slowed with prolonged prophase and metaphase stages, while centrosomes are frequently detached and prematurely separated. As a consequence centrosome and nuclear cycles become uncoordinated, resulting in arrested embryonic development [18], [19]. Microcephaly occurring in MCPH1 primary microcephaly may be due to a defective centrosomal function of microcephalin/BRIT1 influencing the number of proliferative, symmetric cell divisions of neuronal stem cells during neurogenesis.

Many of the accumulating data reported for microcephalin are based on RNAi-experiments. It will be relevant to back up this information by data provided from model organisms. A model system with misregulated chromosome condensation will also be useful to obtain further insights into the molecular mechanisms shaping the mitotic chromosomes.

We present here the first mouse model with defective *Mcph1*-function. The model was generated using a mouse embryonic stem cell line containing a gene trap in intron 12 of the *Mcph1* gene (GeneID 244329) deleting the C-terminal BRCT-domain of the protein. In our study we show that the gene trap mutation impairs the function of the protein resulting in misregulated chromosome condensation in proliferating cells of animals homozygous for the trapped allele.

## Materials and Methods

### Identification of the Gene Trap Insertion Site

Long range PCR kit (Roche) was applied for candidate forward primers designed for 5 Kb intervals of intron 12 of the *Mcph1*-gene. One common reverse primer specific to the vector sequence was applied in all reactions (5′- CTTCCTGTAGCCAGCTTTCATCAACATT-3′). Amplification with forward primers P18 (5′-GAGGAAAAATCTCTCACATCTCCACACG-3′) and P19 (5′- TCAAGTTCACCTCCAACTAAAGGGAAGG–3′) resulted in the amplification of distinct PCR-products of approximately 6.5 kb and 1.5 kb, respectively. PCR conditions were used as follows: 92°C for 10 sec for the first 10 cycles then 15 sec in the next 25 cycles, extension at 68°C for 4 min in the first 10 cycles adding 20 sec per cycle for the next 25 cycles. Final extension was at 68°C for 7 min. Subsequently, the PCR products were analyzed by sequence analysis on a 3730 DNA Analyzer (Applied Biosystems) applying standard procedures.

### Quantitative Real-Time PCR

For relative quantification total RNA was isolated from brain, liver and spleen of 3 Mcph1^gt/gt^ and 3 wild type mice (of same age and generation) using TRIzol (Invitrogen), in accordance with the manufacturer's instructions. First strand synthesis was performed with 2 µg of total RNA using SuperScript III First-Strand Kit (Invitrogen). First strand samples from 50 ng RNA were used for real-time PCRs. qPCRs were carried out using POWER SYBR® GREEN PCR Master Mix (Applied Biosystems) and Applied Biosystems 7500 Real-Time PCR System according to the manufacturer's recommendations. Forty five two-step cycles were performed (15 s at 95°C and 60 s at 60°C). Relative quantification was achieved by the ΔΔCt-method using Mcph1-specific primers F1: 5′-ACTCTCTGAAACCTTCCCTGCAG-3′ and R1: 5′-GGCGCTATGAACATCTTCGG- 3′, F2: 5′-ACTCTCTGAAACCTTCCCTGCAG-3′ and R2: 5′-GGCGCTATGAACATCTTCGG-3′, and Hprt-specific primers F: 5′-TGCTCGAGATGTCATGAAGG-3′ and R: 5′-AATCCAGCAGGTCAGCAAAG-3′.

For absolute quantification the PCR products of Hprt and the wild type and trap alleles of Mcph1 were cloned using the Topo 2.1 Cloning Kit (Invitrogen). The Mcph1-specific primers described above and a primer pair amplifying the Mcph1-trap fusion (F: 5′-TAGAGTTGGGCCACTGGATTTCT-3′ and R: 5′-CTGCGTTCTTCTTCTTTGGTTTTC-3′) were used for PCR cloning. The plasmid concentrations were determined by UV photometry. The plasmids were diluted to generate standard curves from 10^9^ copies/µl to 10^0^ copies/µl. First strand synthesis was performed as described above. First strand samples from 50 ng RNA were used for real-time PCRs. Homogeneous loading was controlled by comparing the Ct-values of β-actin primers F: 5′-CGTGAAAAGATGACCCAGATCA-3′, R: 5′-GGGACAGCACAGCCTGGAt-3′. The respective Ct-values of the Hprt, and Mcph1 wild type and trap alleles were related to the standard curves. The absolute number of target copies per microgram total RNA was calculated and compared in brain, liver, spleen, and fibroblasts between wild type and Mcph1^gt/gt^ mice.

### Generation of the Mcph1 Deficient Mice

The RR0608 ES cells (129/OLA) were microinjected into blastocysts from C57BL/6 mice. Chimeras were identified by coat color and crossed with wild type C57BL/6. Germline transmission resulted in two heterozygote animal, a male and a female, which were crossed inter se. Pairing of heterozygous animals resulted in viable homozygous offspring. All animal experiments were performed according to institutional guidelines. Mice were genotyped by PCR with primers flanking the integration site for the wild type allele (wt-F 5′-AGCCCAGAGCCTAGAGAGGA-3′, wt-R 5′-ATCTGTGCATAGGAGACAACACTGATAG-3′) and a forward primer flanking the 5′-integration site and a reverse primer specific for the trap vector identifying the trapped allele (gt-F 5′-TGCCTATGTGCAATGTTTCAA-3′, gt-R 5′-TGACCTTGGGCAAGAACATA-3′) resulting in PCR products of 989 bp and 181 bp, respectively. PCR conditions for the wild type allele were 96°C for 10 s, 56.5°C for 45 s, 70°C for 1 min 15 s for a total of 35 cycles. The trap allele was amplified by 35 cycles of 96°C for 10 s, 52°C for 45 s, 72°C for 45 s.

### Establishment of Fibroblast Cultures

Mouse tail tips or peritoneum pieces were washed in Earle's Medium containing antibiotics, cut into small pieces and transferred to falcon tubes for trypsin-collagenase treatment. Tail tips were trypsinized for 1 h at 37°C with 0.5% trypsin-EDTA. Subsequently, the supernatant was removed and replaced by 10% collagenase/DMEM for 1 h at 37°C. The resulting cell suspension was washed and transferred to culture flasks. The cells were grown in DMEM supplemented with 15% fetal calf serum and antibiotics.

### Chromosome Preparation and PLC-Assay

Cytogenetic preparations were performed on fibroblasts using standard diagnostic laboratory procedures to prepare metaphase spreads. Slides were stained by immersion in fresh 10% Giemsa stain for 10 min. 2,000 nuclei were counted and percentage of prophase/prophase-like and metaphase nuclei was determined among total nuclei.

### Analysis of Cell Cycle Progression by ^3^[H]-Thymidine Autoradiography

Logarithmically growing mouse fibroblast cell lines were exposed for 10 min to 1 µCi/ml ^3^[H]-thymidine, washed twice with 37°C pre-warmed filtered sterile PBS, and harvested at one-hour intervals (1–4 hours after labeling). The slides were coated with nuclear track emulsion (Ilford) and exposed for one week. Autoradiograms were developed and stained with Giemsa. The labeling patterns of 100 prophases were scored for each time.

### Decondensation Assay

Cytokinesis of primary mouse fibroblasts was blocked with 6 µg/ml of Cytochalasin B for 2 hs before harvesting. Cells were subjected to hypotonic treatment with 0.075 M of KCl for 2 min and were fixed two times with a 3∶1 mix of methanol and acetic acid, and the last fixation was in a 7∶1 mix of methanol and acetic acid. Nuclei were stained with Giemsa. Postmitotic cells were distinguished by their binucleate appearance. Slides were coded and n≥100 binucleated cells were counted from each sample.

### Chromosomal Breakage Analysis

Logarithmically proliferating diploid fibroblast cultures were irradiated using an X-ray source (Muller MG 150, Ua  = 100 kV, I = 10 mA, filter 0.3 mm Ni, dosis rate: 2.1 Gy/min). Chromosomes were prepared at 3 hs and 6 hs after the time of irradiation as described above. Slides were coded and 25 metaphases were scored for each sample at each time point.

### Flow Cytometry

Primary fibroblasts from three Mcph1^gt/gt^ and three wildtype mice were seeded (4.000 cells/cm^2^) in duplicate flasks. The cells were allowed to attach overnight. One culture of each strain was exposed to high-energy X-ray photons provided by a linear accelerator with dosimetry. The dosage level equaled 4 Gy. After another 2 h of incubation, matched irradiated and unirradiated cultures were harvested. The cells were washed in PBS and fixed in 2% para-formaldehyde. Permeabilization was by 90% methanol for 30 min on ice. Phospho-Histone H3 was detected by mouse monoclonal anti p-H3 primary antibody (Cell Signaling Technology, #9706) at a dilution of 1∶25 and goat anti-mouse IgG (H+L) Alexa Fluor 647-conjugated secondary antibody (Molecular Probes, A21237). DAPI was used as counterstain for DNA content and cell cycle distribution. HeCd and Ar laser excitation and fluorescence detection was performed on an analytical flow cytometer (Becton Dickinson, LSRII). p-H3-positive signals were expressed relative to all recorded events.

### Preparation of Meiotic Chromosomes

Meiotic chromosomes were prepared from the testes of adult male mice by conventional chromosome preparation [20]. Briefly, the testes were cut into small pieces. After hypotonic treatment for 15 min at room temperature, the cells were fixed with acetic acid:methanol (1∶3). The cell suspension was dropped onto slides and stained with Giemsa.

### Preparation of Testes Sections

Testes were fixed with Methacarn (60% methanol, 30% chloroform, 10% acetic acid), dehydrated in ethanol, substituted with xylene, and embedded in paraffin. Paraffin sections (5 µm thick) were stained with HE.

### Immunofluorescence

Cells were grown on cover slips to approximately 70% confluency. Cells were fixed with 2% paraformaldehyde in PBS for 30 min. Permeabilization was performed on ice with 0.5% triton X-100/PBS for 5 min. Cover slips were blocked for 1 h in 3% BSA/PBS. Anti- p-H3-S10 (abcam), anti-Aurora B (abcam), anti-53BP1 (Novus), and anti-H2AX-Ser139 (Upstate) were used for immunodetection. Fluorescence staining was performed with secondary antibodies anti-mouse-TRITC (Sigma) and anti-rabbit (SantaCruz). Nuclei were counterstained with DAPI. For the analyses of the formation of DNA repair foci, cell cultures were treated for two hours before fixation with 0.5 µg/ml of the radiomimetic agent adriamycin or 10 µg/ml of the radiomimetic agent bleomycin to induce DNA double strand breaks.

### Magnetic Resonance Imaging

To assess cerebral volume in vivo, wild-type (n = 5), and homozygous Mcph1^gt/gt^ (n = 11) were anaesthetized with 3% isoflurane (Forene, Abbot, Wiesbaden, Germany) mixed with O2 using a Vapor 2000 Vaporizer (Dräger Medical, Lübeck, Germany) and a flowmeter for O2/N2O. During the remainder of the imaging session anaesthesia was maintained with 1.5–2.0% isofluran. To ensure constant body temperature of 37°C mice were placed on a heated circulating water blanket. Respiration was monitored using Small Animal Monitoring & Gating System (SA Instruments, Stony Brook, New York, USA).

Brain images were acquired using a 7 Tesla Bruker rodent scanner (Pharmascan 70/16AS, Bruker BioSpin, Ettlingen, Germany) and a 20 mm Quadrature-Volume-Resonator.

High-resolution T2-weighted images of the whole brain including olfactory bulb and cerebellum were acquired with a 2D turbo spin-echo sequence (TR/TE  = 4059/36 ms, RARE factor 8.4 averages). 35 axial slices with a slice thickness of 0.5 mm, a field of view of 2.85×2.85 cm and a matrix of 256×256 were positioned over the whole brain resulting in an in plane resolution of 111 µm.

### Calculation of Brain Volume

Calculation of brain volume was carried out using Analyze 5.0 software (AnalyzeDirect, Inc.; Lenexa USA). Brains were assigned with a Region of Interest Tool resulting in 3D object maps for each single mouse brain. The volumes of 3D maps were then automatically calculated.

### Brain Proteome Analysis

Control and Mcph1^gt/gt^ mice at 15 weeks of age (n = 4) were sacrificed by cervical dislocation. All brains were immediately removed, weighted, the cortex removed, snap-frozen in liquid nitrogen and stored at −80°C for protein extraction. Total protein extracts were prepared from brain tissue. Frozen tissue, 1.6 parts v/w of buffer P (50 mM TRIZMA Base (Sigma-Aldrich, Steinheim, Germany), 50 mM KCl and 20% w/v glycerol at pH 7.5), supplemented with a final CHAPS concentration of 4% w/v in the sample, 0.08 parts of protease inhibitor solution I (1 Complete tablet (Roche Applied Science, Mannheim, Germany) dissolved in 2 mL of buffer 1) and 0.02 parts of protease inhibitor solution II (1.4 M pepstatin A and 1 mM phenylmethylsulfonyl fluoride in ethanol) were ground to fine powder in a mortar pre-cooled in liquid nitrogen. The tissue powder was transferred into a 2 mL tube (Eppendorf, Hamburg, Germany), quickly thawed and supplied with glass beads (0.034 units of glass beads per combined weight of tissue, buffers and inhibitors in mg; glass beads, 2.5 mm±0.05 mm diameter, Worf Glaskugeln GmbH, Mainz, Germany). Each sample was sonicated 6 times in an ice-cold water bath for 10 s each, with cooling intervals of 1 min 50 s in between. The homogenate was stirred for 30 min in buffer P without CHAPS at 4°C in the presence of 0.025 parts v/w of benzonase (Merck, Darmstadt, Germany) and a final concentration of 5 mM magnesium chloride in the sample. Subsequently, 6.5 M urea and 2 M thiourea were added, and stirring was continued for 30 min at room temperature until urea and thiourea were completely dissolved. The protein extract was supplied with 70 mM dithiothreitol (Biorad, Munich, Germany), 2% v/w of ampholyte mixture Servalyte pH 2–4 (Serva, Heidelberg, Germany), corrected by the amount of urea added (correction factor  =  sample weight prior to addition of urea/sample weight after addition of urea), and stored at −80°C. Protein concentrations were determined in sample aliquots without urea using Biorad DC Protein Assay according to the protocol supplied by the manufacturer.

### Two-Dimensional Gel Electrophoresis

Protein samples were separated by the large-gel 2-DE technique developed in our laboratory as described previously [21]. The gel format was 40 cm (isoelectric focusing)×30 cm (SDS-PAGE)×0.75 mm (gel width). For isoelectric focusing (IEF) using the carrier ampholyte technique, we applied 6 L (∼20 g/L) protein extract of each sample to the anodic end of an IEF-gel and used a carrier ampholyte mixture to establish a pH-gradient from 3 to 10. Proteins were visualized in SDS-PAGE polyacrylamide gels by high sensitivity silver staining. 2-D gels were dried as described [21] and scanned at 300 dpi and 16 bits gray scale using a scanner (Microtek Scan Maker 9800XL, Evestar GmbH, Willich, Germany). The 2-D gel images were subsequently saved in Tiff format to avoid loss of quality due to compression.

After up-loading the 2-D gel images, protein spot patterns were evaluated by Delta2D imaging software version 3.6 (DECODON, Greifswald, Germany) as was already described recently in detail elsewhere [22]. Delta2D is our standard 2-D gel evaluation software and was validated already in many of our studies [23], [24], [25], [26], [27], [28]. Briefly, 2-D spot patterns of Mcph1^gt/gt^ and control mouse brains were matched using the Delta2D “exact” mode matching protocol. First sample pairs (Mcph1^gt/gt^ and control) were matched individually. Subsequently, all Mcph1^gt/gt^ gels were matched to create a “match link” between all 2-D spot patterns using match vectors [22]. Using “union” mode a fusion image was generated, including the visible spots for each 2-D gel. Only the fusion image was used for spot detection employing the following settings for Delta2D: local background region: 100, average spot size: 1, sensitivity: 100%. Spots were not edited manually after spot detection. About 2,000 spots on the fusion image were transferred to all other 2-D gel images for each time point. This ensures that for each stage investigated the ID for each spot on a gel is identical. Relative spot volume intensities (fractions of 100%) were used for quantitative protein expression analysis. After background subtraction, normalized spot intensity values were copied into Excel spreadsheets for statistical analysis. Data sets were analyzed applying a paired Student's t-Test.

### Expression Profiling

Total RNA was isolated from brain, liver and spleen of 3 MCPH1^gt/gt^ and 3 wild type mice (of same age and generation) using TRIzol (Invitrogen), in accordance with the manufacturer's instructions. Aliquots of RNA samples (500 ng) were amplified according to the specifications of the Illumina Total Prep RNA Amplification Kit (Ambion, Austin, TX, USA). The cRNA samples were applied to the arrays of Sentrix^®^ Mouse-6 Expression BeadChip (Illumina, San Diego, CA, USA). Signal was developed with streptavidin-Cy3, and the Sentrix BeadChip was scanned with the Illumina BeadArray Reader. The DiffScore parameters were used to determine gene expression in the Illumina BeadStudio software program (v3.1.3.0). We utilized the rank-invariant method algorithm provided by BeadStudio Gene Expression Module for normalization of sample signals.

## Results

### Characterization of the Gene Trap in the ES Cell Line RR0608

The gene trap vector used by BayGenomics contains a splice-acceptor sequence upstream of a reporter gene (β–geo, a fusion of β–galactosidase and neomycin phosphotransferase). The insertion of the vector into an intron of a gene results in a fusion transcript that joins the sequence of the exon lying 5′- to the insertion site and the β-geo reporter resulting in a truncated traceable fusion protein [29]. In the ES cell line RR0608 the gene trap vector inserted between exons 12 and 13 of the *Mcph1* gene as identified by 5′RACE (BayGenomics database, http://baygenomics.ucsf.edu, now moved to http://www.genetrap.org/) resulting in a truncated protein lacking 97 amino acids in the most C-terminal BRCT-domain. The mutant allele will be referred to as Mcph1^gt^. The truncated transcript was characterized by RT-PCR using gene and trap-specific primers and subsequent sequencing. Sequencing of the PCR product confirmed the fusion of *Mcph1* exon 12 and the reporter.

**Figure 1:**
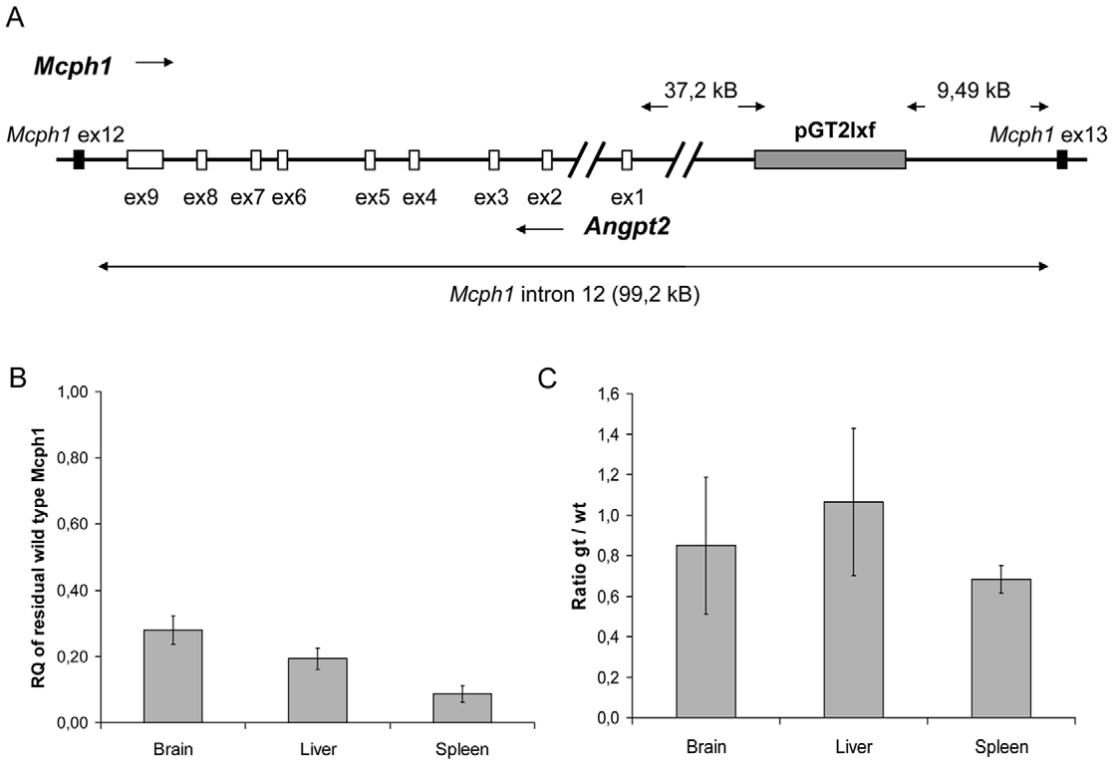
Characterization of the gene trap allele. (A) The vector was inserted into intron 12 of the *Mcph1* gene. The gene *Anpt2* is localized on the opposite strand within this intron. Flanking *Mcph1* exons are displayed in black, *Angpt2* exons are displayed in white and the vector is displayed as grey box. The vector inserted 37.2 kb upstream of exon 1 of *Anpt2* and 9.49 kb upstream of exon 13 of *Mcph1*. (B) Relative quantification of residual wild type Mcph1-mRNA in tissues of Mcph1^gt/gt^ animals by real-time PCR. The expression of wild type Mcph1-mRNA is significantly reduced in all analyzed tissues, although a considerable residual amount of wild type Mcph1-mRNA is detected. (C) The expression levels of the β-geo-fusion-mRNA compared to residual wild type Mcph1-mRNA in different tissues of Mcph1^gt/gt^ animals expressed as the ratio of β-geo-fusion-mRNA to wild type Mcph1-mRNA. Wild type and β-geo-fusion-mRNA are expressed at roughly the same low levels. The absolute numbers of mRNA copies per microgram RNA were determined by quantitative PCR.

It was crucial to identify the genomic insertion site, as the gene *Angpt2* resides in intron 12 of the *Mcph1* gene (Figure 1). Thus, the insertion of the gene trap may have disrupted *Angpt2* which is a lethal condition in mouse [30]. For this purpose we subdivided the intron (intron size is 99,179 bp) into sections of approximately 5,000 bp by designing sequence specific forward primers. We then tried to achieve PCR amplification by long range PCR using a trap specific reverse primer. PCR using primer P19 (5′-TCAAGTTCACCTCCAACTAAAGGGAAGG-3′) resulted in the amplification of a 1,500 bp product. Subsequent sequencing of the PCR product determined the insertion site of the trap at the position 18,778,752 of the genomic sequence (USCS; version July 2007) c.2,176-9,486pGT2lxf, therefore, in a distance of approximately 37,200 bp of exon 1 of the *Angpt2* gene (Figure 1A).

Quantitative real time PCR confirmed that the expression of the *Mcph1* wild type transcript is markedly reduced in different tissues of Mcph1^gt/gt^ mice. Primer pairs spanning introns 12 or 13 were used for the amplification of the *Mcph1* wild type transcript. The relative quantification was achieved applying the ΔΔCT method. Normalization was reached using Hprt as endogenous reference gene, and RNA from wild type animals was used for standardization. We compared the expression in liver, brain, and spleen of three Mcph1^gt/gt^ animals to three controls by relative quantification. In Mcph1^gt/gt^ the mRNA level of wt *Mcph1* was reduced to approximately 9%±2.5 in spleen, 19%±3.2 in liver, and 28%±4.3 in brain, indicating that the *Mcph1* genetrap mutation results - as expected - in a preferential splicing of exon 11 of the *Mcph1*-gene to the gene trap vector. Obviously, however, alternative splicing allows a strongly reduced but still detectable expression of the wild type allele and this expression varies between different tissues (Figure 1B).

Residual wild type protein might be able to exert some function, however with significantly diminished activity due to its reduced abundance. In addition, even the trap-allele may adopt some Mcph1-functions because the genetrap truncates only the C-terminus of the protein. Therefore, we intended to further characterize the expression from both the wild type- and trap-allele. Unfortunately, we were not able to detect the wild type Mcph1 protein by western blotting neither in protein samples from wild type nor Mcph1^gt/gt^ animals. Also the β–geo reporter was not traceable in Mcph1^gt/gt^ neither by X-gal staining nor by anti-β–galactosidase antibodies. This may be explained by low expression rates of Mcph1. Therefore, we decided to perform an absolute quantification of the mRNAs of both alleles and an endogenous control gene (*Hprt*) in the different tissues by real time PCR. The results revealed that in comparison to the low-abundant endogenous control gene *Hprt, Mcph1* is expressed at very low levels in all analyzed tissues. In wild type tissues the copy numbers of Mcph1 per microgram RNA compared to Hprt were 3.7±1.6 times less in spleen, 10.7±1.2 times less in liver, and 21.6±3.7 times lower in brain and accordingly even lower in the tissues of the Mcph1^gt/gt^ animals (76.9±21.1 times, 45.1±12.1 times, 46.3±3.6, respectively). Thus, our problems detecting the Mcph1 protein can be explained by these very low expression levels. Surprisingly, the analyses also revealed that the gene trap allele is expressed at approximately the same low level as the remaining wild type allele in the tissues of the Mcph1^gt/gt^ animals (the ratios of expressed gene trap to wild type allele were 0,85 in brain, 1,07 liver, and 0,68 in spleen, Figure 1c). Therefore, the addition of the β-geo cassette may either interfere with the regulation of gene expression or destabilize the mRNA expressed by the trap-allele. Thus, the low expression level not only may explain our problems detecting the reporter protein but also infers that it may not be possible for the fusion protein to substantially adopt Mcph1-functions.

### Phenotypic Description of the Mice

**Figure 2:**
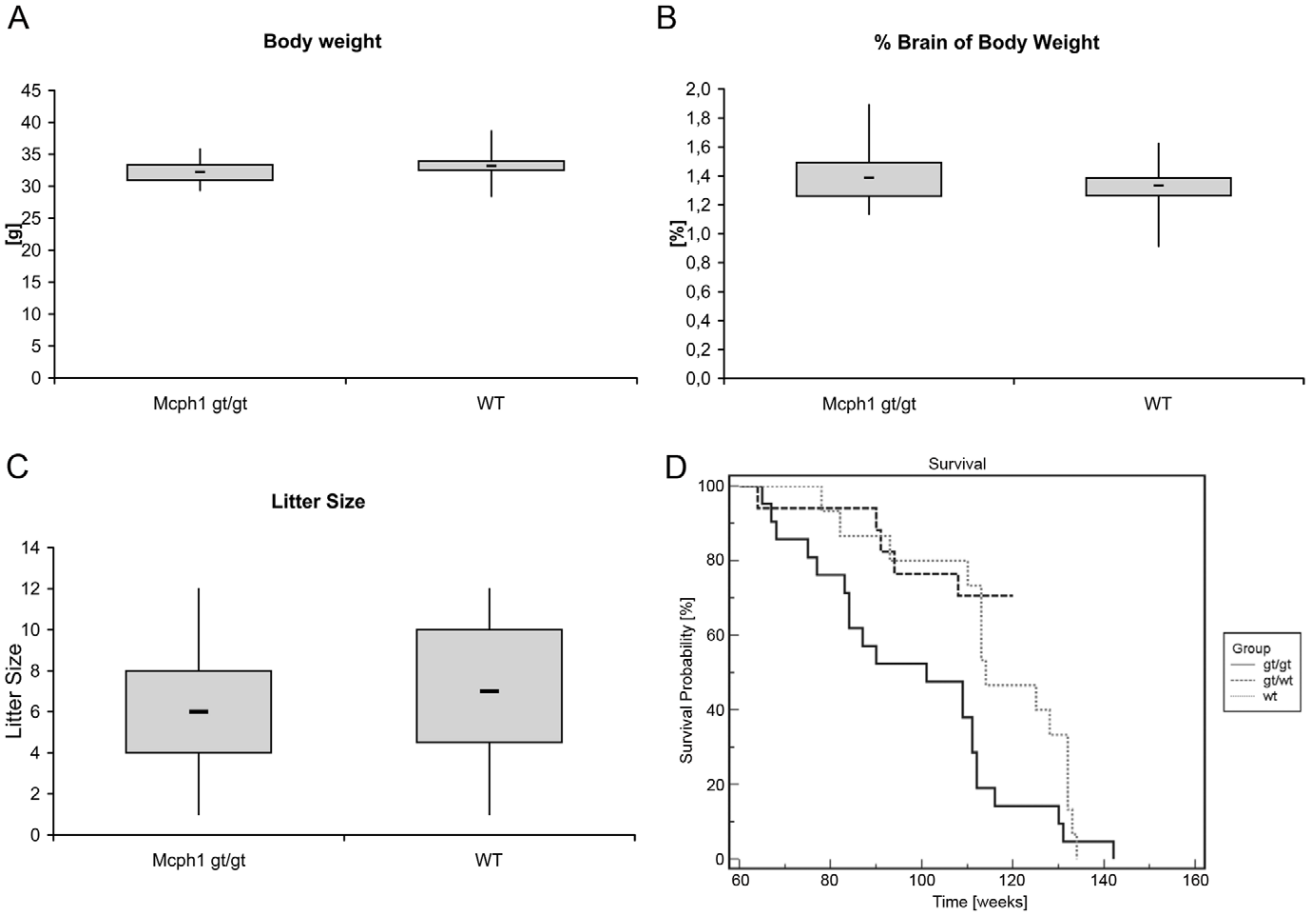
Evaluation of the physical phenotype of Mcph1^gt/gt^ mice. Boxplots showing that Mcph1^gt/gt^ mice do not differ significantly from wild type animals concerning body weight (P = 0.193) (A) and the proportionate weight of their brains (% brain of body weight, P = 0.275) (B). Mcph1^gt/gt^ mice do not show a reduction in fertility (P = 0.441) (C). Kaplan-Meier survival curves of Mcph1^gt/gt^ compared to wild type mice show significantly reduced survival rates of Mcph1^gt/gt^ compared to wild type and heterozygote animals (D).

**Figure 3:**
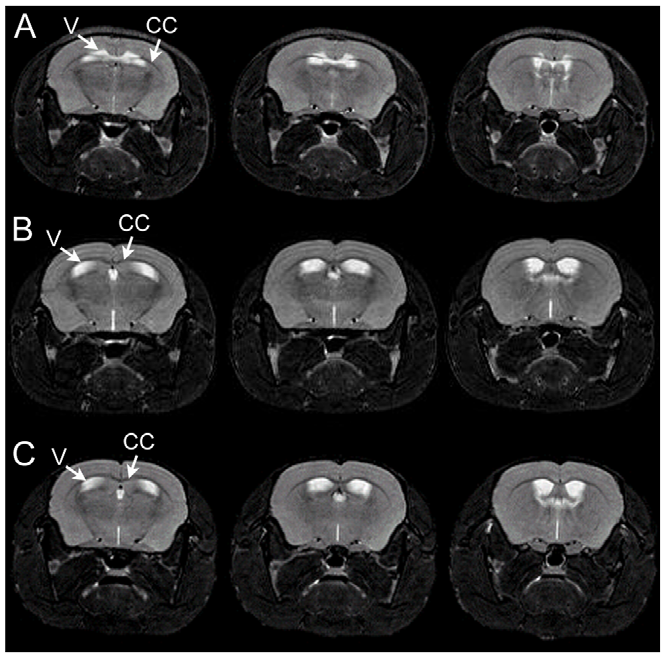
T2-weighted MR images of Mcph1^gt/gt^ mouse brains. (A) Different brain sections of the single Mcph1^gt/gt^ mouse presenting with hypoplasia of the corpus callosum (dark line, CC) and ventricular (light structures, V) malformation. (B) The brains of the majority of Mcph1^gt/gt^ mice showed normal forebrain anatomy and did not differ from those of WT animals. (C) Wild type mouse with normal forebrain anatomy.

Mcph1^gt/gt^ animals did not differ significantly in body weight compared to wild type animals (mean 32.3 g±1.8, n = 14 vs. 33.5 g±2.6, n = 12, respectively, unpaired t-testing results in a two-tailed P-value P = 0.193) (Figure 2A). By conventional criteria this difference is not considered to be statistically significant. Equally, the proportionate weight of the brains of Mcph1^gt/gt^ animals was not reduced as compared to wild type (mean 1.4%±0.21, n = 14 vs. 1.32±0.16, n = 12, respectively, P = 0.275), (Figure2B). Measurement of brain volumes by MRI supported these findings. The average volume of Mcph1^gt/gt^ brains (n = 11) was 522.5 mm^3^±22.8 compared to 514.4 mm^3^±10.4 in wild type animals (n = 4). Moreover MRI analyses did not reveal any obvious malformations of the brain (Figure 3). However, in one of the 11 Mcph1^gt/gt^ animals aplasia of the corpus callosum and atrophy of the 3^rd^ ventricle was found. Ventricular malformation indicates a midline defect caused by a developmental disorder of the forebrain (Figure 3A). This is rarely observed in mice, but as it represents a single event, it must be rated anecdotal, as long as no further incidences are found.

Most importantly however, the overall survival is reduced compared to wild type and heterozygous animals. 21 Mcph1^gt/gt^, 17 heterozygous and 15 wt animals were maintained under standard conditions to examine survival. The difference in survival develops after animals have reached an age of >65 weeks and is statistically significant for the comparison of Mcph1^gt/gt^ and wt animals (log rank test χ^2^ = 4.1818, P = 0.0409) as well as the comparison of Mcph1^gt/gt^ and heterozygous animals (log rank test χ^2^ = 10.1002, P = 0.0015, Figure 2D). Unfortunately, we could so far not determine the precise causes of deaths for the Mcph1^gt/gt^ animals.

### Analyses of Chromosome Condensation

**Figure 4:**
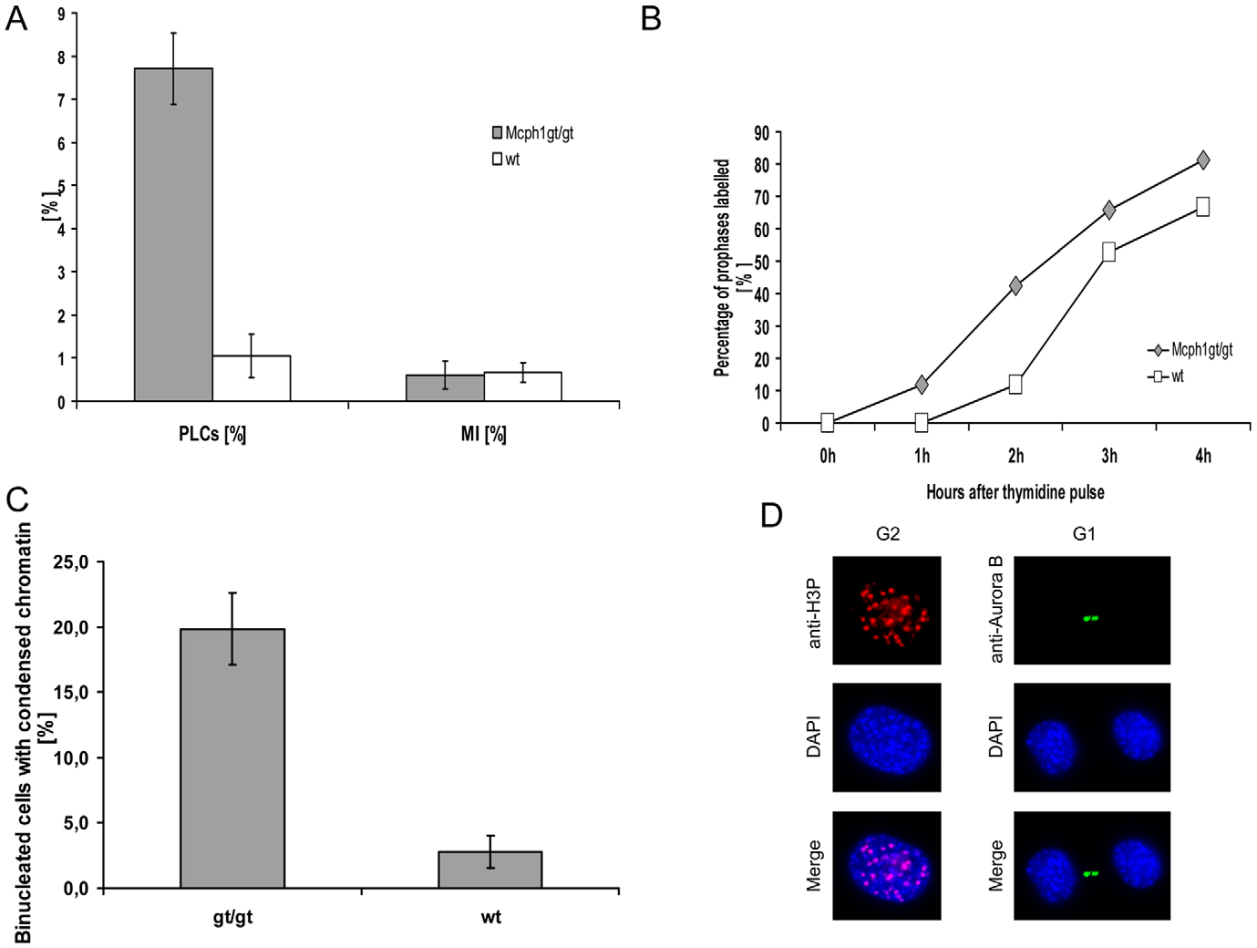
Analyses of chromosome condensation behavior in Mcph1^ gt/gt^ cells. (A) Determination of the fraction of prophase-like cells (PLCs) and metaphase indices in Mcph1^gt/gt^ and WT cell cultures. Mcph1^gt/gt^ show significantly increased proportions of PLCs: 7.7% vs. 1.0% in WT cell cultures (χ^2^ = 1306.49, p<0.001). In contrast, metaphase indices (MI) are within the range of wild type cell cultures, indicating comparable proliferative activity. (B) Cell cycle analysis after 3H-thymidine-pulse-labeling of logarithmically growing fibroblast cell lines from Mcph1^gt/gt^ (grey diamonds) and WT animals (white squares). The first labeled cells with prophase-like appearance in the Mcph1^gt/gt^ cells occur within the first hour after the pulse. (C) Proportion of cells with condensed chromosomes postmitosis in Mcph1^gt/gt^ and control cells. Many Cytochalasin-treated binucleated G1-cells of Mcph1^gt/gt^ cells show condensed chromatin (19.8%) compared with control cells (2.8%). (D) To the left one exemplary cell displaying premature chromosome condensation in the G2 phase of the cell cycle defined by focal (centromeric) staining of phosphorylated histone H3(-Ser10), note that the phosphorylation has not spread into the chromosome arms. To the right two cells showing delayed decondensation in G1 post-mitosis defined by the formation of a midbody.

Primary fibroblast cultures from 11 Mcph1^gt/gt^ mice and 12 wild type animals were established from mouse tails and propagated under standard conditions. Cytological preparations were made in parallel from those 11 Mcph1^gt/gt^ and 12 Mcph1^wt^ cultures to analyse chromosome morphology. The slides were coded and the fraction of prophase-like cells (PLCs) was determined by visual cell counts. 2,000 nuclei were counted from each sample and the proportion of nuclei with prophase-like morphology and the metaphase indices were determined. Mcph1^gt/gt^ cell lines showed a dramatic increase of nuclei displaying condensed prophase-like chromatin (7.7%±0.8) compared to wild type cell lines (1.0%±0.5, χ^2^ = 1306.49, p<0.001) (Figure 4A). In contrast, the metaphase indices were at the same level: 0.6%±0.3 and 0.7%±0.2 for gt/gt and wt cell lines, respectively. The similar metaphase indices confirm similar proliferative activities in gt/gt and wt/wt cell cultures. Quantitative analyses in 5 heterozygous cell lines revealed no increase in the proportion of prophase-like cells (1.22%±0.5), an observation arguing against a potential dominant-negative effect of the gene trap allele.

We determined the timing of chromosome condensation following S phase by ^3^[H]-thymidine pulse labeling. For this purpose, we compared one logarithmically growing diploid fibroblast cell line and one transformed cell line from Mcph1^gt/gt^ mice to wild type cell lines in 1 h intervals (1 h–4 h) by autoradiography after pulse-labeling with ^3^[H]-thymidine for 10 min. N≥100 prophases or prophase-like cells were scored per time point and per cell line and the proportion of labeled cells was determined. We observed a significant difference between the gt/gt and wt cell lines in the proportion of prophase/prophase-like cells that were labeled within this period. Already 1 h after the pulse, 12% of the prophase-like cells of the diploid gt/gt mouse but none of the prophase cells in the control showed 3H-thymidine labeling. After 2 h, 42% of PLCs in the diploid Mcph1^gt/gt^ cells and 12% of the prophases in wt cells were labeled (see Figure 4B). Similar results were obtained for the transformed cell lines. 1 h after the pulse 35% of PLCs were labeled in the Mcph1^gt/gt^ cell line compared to only 12% in the wild type (Figure S1). Consequently, chromosome condensation in the Mcph1^gt/gt^ cells starts in the early G2 phase of the cell cycle as soon as 1 h after the end of the S-phase or even earlier. Thus, the observed premature chromosome condensation phenotype described in the cells of human patients with *MCPH1*-deficiency is also found in mouse cells homozygous for the trap-allele.

To investigate the chromosome decondensation behavior of the Mcph1^gt/gt^ cell lines in the early G1 phase of the cell cycle we treated three logarithmically growing Mcph1^gt/gt^ fibroblast cell lines and three wild type fibroblast cell lines with the inhibitor of cytokinesis Cytochalasin B. Treatment with Cytochalasin B results in binucleated cells. Cytological preparations were performed following 2 hs of Cytochalasin treatment. Consequently, all binucleated cells must have exited mitosis within these two hours and, therefore, be in early G1 phase. 19.8% (± 2.7) of binucleated Mcph1^gt/gt^ cells displayed condensed chromatin, while only 2.8% (± 1.2) of wild type binucleated cells showed signs of chromatin condensation, (χ^2^ = 84.32, p<0.001) (Figure 4C). Thus, there is a delayed decondensation in Mcph1^gt/gt^ cells.

These data were supported by immunofluorescence analyses. In normal prophases phosphorylation of histone H3 at serine 10 (p-H3) usually has spread to the chromosome arms while human MCPH1 cells show prophase like cells in the G2 phase of the cell cycle without full phospho-histone H3 labeling (Alderton et al, 2006). Similarly, we observed high numbers of equivalent cells in Mcph1^gt/gt^ fibroblast cultures. Figure 4D shows a cell displaying clearly condensed chromatin with only centromeric p-H3-labeling. These cells are accordingly in G2 and show prophase-like condensation but are different from normal prophases on a molecular level, because in normal prophases histone H3-phosphorylation has spread out over the whole chromosome arms (see Figure S2 for an exemplary normal prophase cell). Pairs of post-mitotic cells can be defined by the formation of a midbody between daughter cells. Following midbody staining with antibodies against Aurora B kinase, we observed a high proportion of post-mitotic cells with clearly condensed chromatin (Figure 4D). Therefore, these cells show delayed chromosome decondensation post-mitosis equal to cultured human fibroblasts with *MCPH1* mutations [7]. In summary, these results confirm a defective chromosome condensation in the Mcph1^gt/gt^ cells similar to MCPH1-deficiency in human.

### Meiosis and Fertility

**Figure 5:**
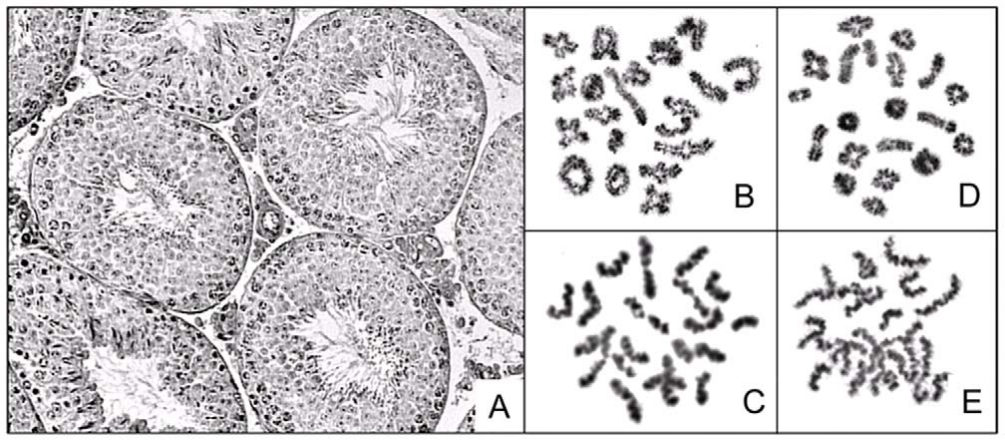
Meiosis in male Mcph1^gt/gt^ germ cells. (A) Section of a testes biopsy from a male Mcph1^gt/gt^ showing normal morphology, (B) diakinesis/metaphase I from a male Mcph1^gt/gt^ reveals normal pairing of the autosomal bivalents and the sex chromosomes (XY), (C) normal metaphase II cell from a male Mcph1^gt/gt^. For comparison a diakinesis/metaphase I (D) and a metaphase II cell (E) from a male wt/wt mouse are presented.

We were interested whether the gene trap mutation may also perturb meiosis and in particular meiotic chromosome condensation. Therefore, male meiosis was investigated by the analyses of testes sections and conventional chromosome preparations. Testes sections show perfectly normal morphology with numerous mature sperm in the lumen. More than 20 metaphase I cells were investigated all of which showed perfectly normal pairing of the homologues. Thus, the analyses of male meiosis revealed normal progression through both meiotic divisions and the production of mature gametes (Figure 5).

In addition, pairing homozygous Mcph1^gt/gt^ mice inter se revealed no reduction in fertility. Average litter size of gt/gt animals was 6.3±2.8 which is not significantly different from that of wild type animals 6.9±2.9 (two-tailed P-value, P = 0.441) (Figure 2C). Likewise, we could not identify any sex specific differences in the breeding success.

### DNA Damage

Several reports attribute a crucial function in DNA damage response to microcephalin/BRIT1. Xu et al. (2004) and Lin et al. (2005) observed that downregulation of MCPH1 by RNAi impaired the ionizing radiation-induced G2/M checkpoint response in U2OS osteosarcoma cells. In contrast, accurate G2/M checkpoint function had previously been reported in *MCPH1*-mutated patient-derived lymphoblastoid cell lines [4]. In order to examine the DNA-damage induced G2/M checkpoint response in Mcph1^gt/gt^ mouse fibroblasts, phospho-histone H3 (Ser^10^) (p-H3) expression was studied as a mitotic marker. Using G2 phase irradiation under exactly identical conditions as described by Xu et al. (2004), flow cytometric analysis revealed a decrease of p-H3-positive Mcph1^gt/gt^ fibroblasts by 87.2% 2 h after provision of 4 Gy. For comparison, wildtype mouse fibroblasts treated in the same way showed a reduction of p-H3-positive cells by 81.2%. In triplicate experiments, these figures were not statistically significantly different (single-tailed t-test, p = 0.189). The corresponding figures 4 h after exposure towards 4 Gy were 86.8% p-H3 reduction in Mcph1^gt/gt^ fibroblasts and 79.6% reduction in wildtype fibroblasts (single-tailed t-test, p = 0.200). Therefore, decreased *Mcph1* expression in Mcph1^gt/gt^ mouse fibroblasts is not associated with defective G2/M checkpoint functions. These data show that Mcph1^gt/gt^ mouse fibroblasts can efficiently prevent mitotic entry despite the gene trap mutation.

**Figure 6:**
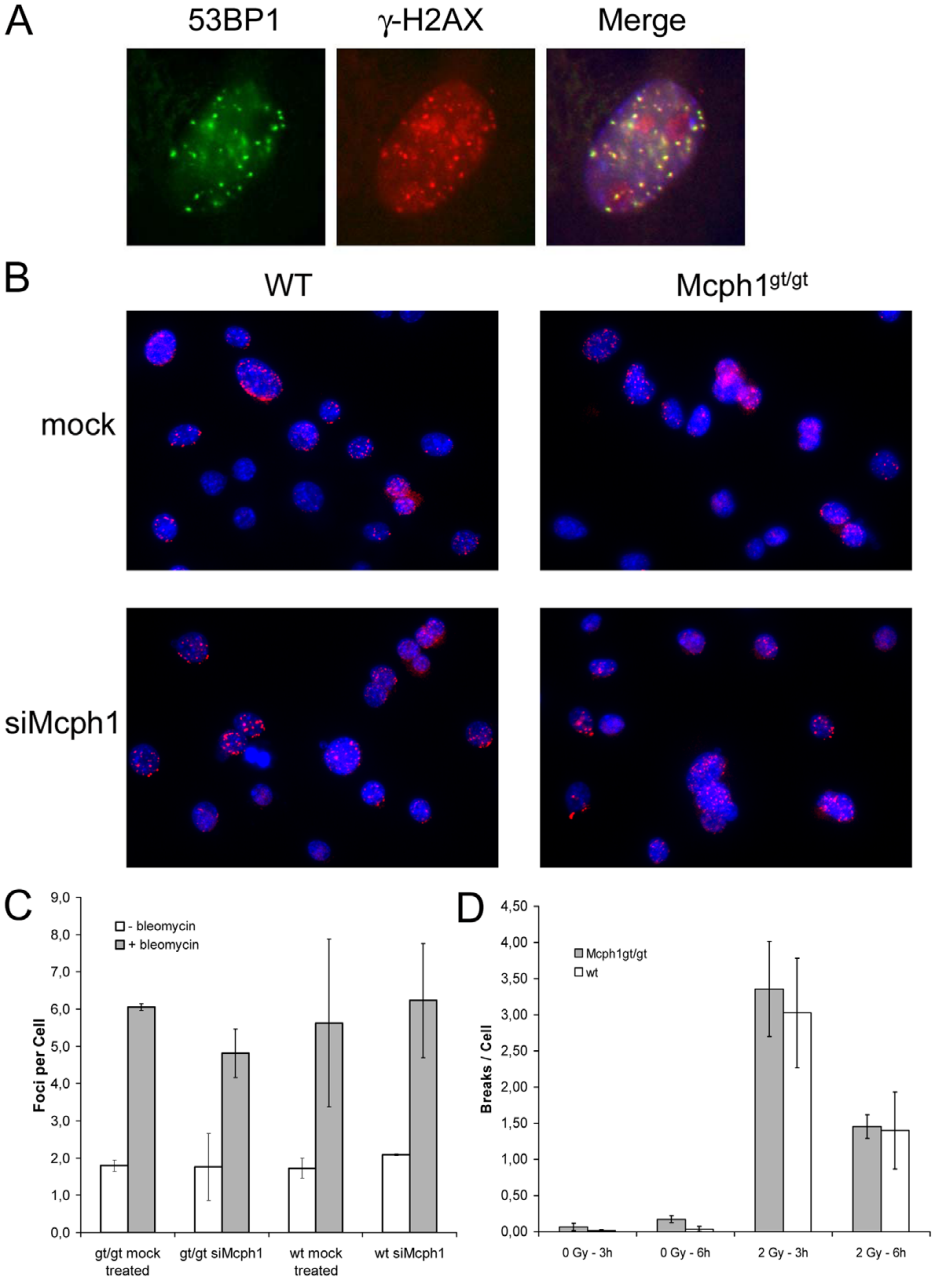
DNA damage response in Mcph1^gt/gt^ cell culture. (A) 53BP1 (green) localizes to repair foci in Mcph1^gt/gt^ cells induced by treatment with the radiomimetic drug adriamycin. The foci co-localize with γ-H2AX foci (red) confirming that the focal staining represents indeed repair foci at the sites of DNA double strand breaks. (B) Overview pictures of 53BP1 foci formation (red) in wild type and Mcph1^gt/gt^ cells following the induction of DNA double strand breaks by the radiomimetic drug bleomycin and treatment with Mcph1-siRNAs (bottom) and mock treatment (top). No obvious difference is detectable in the Mcph1^gt/gt^ cells or siRNA treated cells compared to the controls. (C) Quantitative analyses of the same experiments. No significant difference in the formation of 53BP1 foci in the Mcph1^gt/gt^ cells or siRNA treated cells compared to the controls could be detected. (D) Chromosomal breakage rates in Mcph1^gt/gt^ and wt cells following ionizing irradiation with 2 Gy. There is no significant difference between wild type and Mcph1^gt/gt^ cells.

Moreover, it was reported that microcephalin function is essential for targeting DNA damage response proteins such as 53BP1 to DNA repair foci at the sites of DNA double strand breaks (Rai et al. 2006). The targeting of microcephalin/BRIT1 itself to the repair foci was assigned to the C-terminal BRCT-domains (Wood et al. 2007; Jeffers et al. 2008). Therefore, we explored whether the gene trap deleting the last C-terminal BRCT domain interfered with foci formation following DNA double strand break induction. In contrast to the reported results, the formation of 53BP1 foci that co-localized to γH2AX-foci seemed not disturbed in diploid fibroblast cell cultures of Mcph1^gt/gt^ mice following 2 hours treatment with 0.5 µg/ml of the radiomimetic agent adriamycin (Figure 6A). We did not observe any obvious difference in the formation of repair foci compared to wild type cell cultures. Possibly, this may be explained by residual wild type Mcph1 protein. Also the truncated protein produced by the gene trap allele may be sufficient to enable 53BP1 foci formation. Therefore, we repeated the experiment with cultures of transformed fibroblasts of wild type and Mcph1^gt/gt^ mice and depleted the cells of both Mcph1 wild type and Mcph1^gt^ alleles by RNAi. Quantitative real time PCR showed that Mcph1 and Mcph1^gt^ were efficiently depleted by >70% (Figure S3). Determination of the number of prophase-like cells confirmed that the depletion was efficient enough to induce the cellular phenotype of misregulated chromosome condensation (Figure S3). In wild type cells the number of prophase-like cells increased from 2.0%±0.3 to 11.8%±1.6 after siRNA treatment while the percentage of PLCs was not increased in Mcph1^gt/gt^ cells (11.1%±2.6 in the mock treated cells, 12,1%±2,2 in the siRNA treated cells). Again, there was no obvious difference in the formation of 53BP1-foci between wild type and Mcph1^gt/gt^ cells following the induction of DNA double strand breaks for 2 hs with 10 µg/ml bleomycin. Surprisingly, there was also no obvious difference between RNAi treated and mock treated cells neither in wt nor in Mcph1^gt/gt^ cells (see Figure 6B for overview pictures of the different preparations). To exclude a more subtle effect the different preparations were analyzed quantitatively. Several overview pictures were taken randomly from different areas of each preparation. The pictures were coded and foci numbers in >100 cells were scored for each sample from these coded pictures of single focal planes by four different evaluators in two experiments. Also the quantitative analysis revealed no difference between Mcph1^gt/gt^ and wild type cells. As well, there was no difference in foci numbers between RNAi treated and mock treated cells (Figure 6C).

It was also reported that RNAi against MCPH1 results in chromosomal aberrations [14]. However, we do not observe an increase in chromosomal breakage rates in cells of Mcph1^gt/gt^ mice. Induced chromosomal breakage rates of Mcph1^gt/gt^-cells following ionizing irradiation (2 Gy) were not different from those of wild type cells treated with the same dose (Figure 6D). Three gt/gt cell lines were compared to three wt cell lines. Chromosomes were prepared 3 h and 6 h after irradiation. The number of breaks was scored for 25 metaphases of each cell line at each data point. Unirradiated cell cultures were treated in exactly the same way as their irradiated counterparts. Combined breakage rates for all gt/gt-cultures were 3.36 breaks/cell ±0.66 (3 h after irradiation with 2 Gy) and 1.45 breaks/cell ±0.16 (6 h after irradiation with 2 Gy). The corresponding breakage rates for the wt-cultures were 3.03 breaks/cell ±0.76 and 1.40 breaks/cell ±0.53, respectively.

### Effects on Gene Expression

It was repeatedly reported that *MCPH1* would regulate protein and transcript levels of other genes such as *hTERT*, *BRCA1* and *CHK1* [11], [12], [31], [32]. Therefore, it was speculated that microcephalin/BRIT1 may function in transcriptional regulation [33]. Very recently an interaction of microcephalin/BRIT1 with the transcription factor E2F1 was described [34]. In this study, the C-terminal BRCT-domains were identified to be crucial for E2F1 binding and activation. Therefore, we investigated the effects of the gene trap mutation on gene expression in different tissues on RNA and protein level. We performed expression analyses using Sentrix^®^ Mouse-6 Expression BeadChip (Illumina, San Diego, CA, USA) comparing gene expression in brain, liver, and spleen of 3 Mcph1^gt/gt^ and 3 wildtype animals. (All information including the raw data can be downloaded from http://ws.molgen.mpg.de/ws/311013/MIAME.rar.) These analyses revealed no specific patterns of differentially regulated genes in the animal bearing the gene trap. In particular, in none of the three analyzed tissues did we find an enrichment of regulated genes in any of the pathways microcephalin/BRIT1 is known to be involved. (See Tables S1 and S2 (A-C) for the whole lists of genes differentially expressed at a significance level of P≤0.05, comparing tissues from Mcph1^gt/gt^ and wild type animals). Combining the data of all three tissues resulted in 39 genes commonly upregulated and 23 genes commonly downregulated with a P-value ≤0.05 in all three tissues of the Mcph1^gt/gt^ animals investigated (Figure S4 and Tables S1 and S2 (D-G)). We were unable to assign a specific function to these groups of genes. Interestingly, in all tissues we found more genes up- than downregulated. This was somewhat surprising given the reported role of microcephalin/BRIT1 in transcriptional activation [34]. More specifically, there was no significant reduction in the expression levels of Chk1, Brca1, Topbp1, Rad51, Ddb2, p73, Apaf1, or caspases which were all described to show reduced expression following *MCPH1* knockdowns [34] (Tables S3, S4, S5).

In our proteomic investigation of Mcph1^gt/gt^-brains we detected 4,006 protein isoforms. A total of 63 protein isoform alterations were found when comparing Mcph1^gt/gt^ to control brains. In contrast to the expression profiling, in this assay more protein isoforms (52) appeared to be downregulated than upregulated (11) (Table S6). Comparing the Mcph1^gt/gt^ data to proteomics studies of three neurodegenerative disorders (ND) it became obvious that the number of changes occurring in the brain of gt/gt mice, however, are a lot less numerous than in ND. We investigated mouse models of Huntington's- (HD) [28], Alzheimer's- (AD) [24] and Parkinson's disease (PD) [27]. In the brains of these model organisms a maximum of 240, 82 and 153 protein isoforms were altered compared to wild type animals, respectively. 4,283, 1,769 and 3,293 protein isoforms were studied for each ND, respectively. Correcting for the difference we obtained 5.6%, 4.6% and 4.6% of isoforms altered for HD, AD and PD. In Mcph1^gt/gt^-brains, however, only 1.6% of all protein isoforms were altered. This is about 2.5 fold lower when compared to the neurodegenerative diseases described above. The lower number of changes correlates with a very mild phenotype found in MCPH1^gt/gt^ mice.

## Discussion

We describe here the first mammalian model with misregulated mitotic chromosome condensation due to defective *Mcph1* function. The gene product of the mouse *Mcph1* gene is truncated by a gene trap within intron 12 of the gene which results in the deletion of the last BRCT-domain of the protein. The observed defect in the regulation of chromosome compaction resembles that observed in human PCC syndrome. The chromatin condenses prematurely in the G2 phase of the cell cycle, shortly after completing replication. Post-mitosis the decondensation of the chromatin is delayed. This results in an elevated fraction of cells with condensed chromatin displaying a prophase-like appearance. This unique cellular phenotype proves that the protein function is impaired although the gene trap results in a deletion of only 97 C-terminal amino acids. In addition, our data suggest that the addition of the β-geo reporter interferes with normal function and/or folding of the protein. In this context it seems noteworthy that in contrast all germ line mutations in human *MCPH1* reported so far affect the N-terminal part of the protein. Also, it was recently reported that it is indeed the N-terminal BRCT-domain that is required for the regulation of chromosome condensation in mouse embryonic fibroblasts [17]. Our results, however, suggest that the whole Mcph1 protein may be necessary for an unperturbed function in the regulation of chromosome condensation. The observation that the defective chromosome condensation occurs only in the homozygous but not the heterozygous cell lines makes a dominant-negative effect exerted by the β-geo-reporter unlikely.

Despite the defect in chromosome condensation due to the obviously impaired function of the protein, the Mcph1^gt/gt^-mice do not show any striking physical phenotype. This may not be really surprising, as the major clinical symptom of the human disorder is the severe microcephaly due to a marked reduction in the size of the cerebral cortex [5], [6]. The reduction of the cerebral cortex, however, does not result in significant neurological dysfunction apart from a moderate reduction in cognitive ability [8]. Evolutionary, the enormous encephalization that has occurred in the lineage leading to modern humans is mainly reflected in an increase of the cortical size of the human brain. Therefore, a defect in neurogenic mitosis, which is probably the reason for the microcephaly in humans bearing *MCPH1* mutations, may have only a marginal impact on the formation of mouse brain, that could remain undetected by the methods applied. In this context it should also be noted that the residual expression of the wild type allele was highest in brain (approximately 28%) and it is entirely conceivable that the residual protein allows a normal brain development. Also *Mcph1* isoforms that may be unperturbed by the gene trap mutation could substitute for the function of the truncated protein.

What seems to be more surprising is that meiosis appears to be undisturbed by the defect. At least, we did not observe any limitation in the reproductive capacity of gt/gt mice of both sexes and the morphology of meiosis I spreads of male Mcph1^gt/gt^ mice was normal. Therefore, microcephalin/BRIT1 may either not function in meiotic chromosome condensation or proper meiotic function may not be harmed by an abnormal condensation behavior (undetectable in our preparations). Previously, we have shown that the condensation defect in human cells has no impact on the proper execution of the main mitotic function, that is the segregation of sister chromatids to the daughter cells [4]. However, meiotic division may be more susceptible. Therefore, it will be interesting to analyze the meiotic behavior of chromosomes in Mcph1^gt/gt^ mice in more detail.

While, to the best of our knowledge, there are no reports of associated diseases in MCPH1 patients, we observed a significantly shortened overall lifespan of Mcph1^gt/gt^ animals (Figure 2D). It will be very important to further investigate the effects of the mutation on the health of the animals and its potential relevance for the clinical prognosis of human MCPH1 patients. Unfortunately, we were so far unable to delineate the reasons for the premature deaths in the Mcph1^gt/gt^ animals. But it is tempting to speculate that the shortening of the lifespan is linked to the reported crucial role of microcephalin/BRIT1 in DNA damage response [33], [35].

It was reported that RNAi directed against microcephalin/BRIT1 in the human U2OS cell line results in increased chromosomal aberration rates and prevents foci formation of important regulators of the DNA damage response, such as 53BP1. Importantly, several studies assign the decisive role in the execution of the DNA damage response functions of the protein to the C-terminal BRCT-domains [14], [15], [16]. Deletion of the C-terminal BRCT-domain alone is sufficient to abolish the ability of microcephalin/BRIT1 to form radiation induced foci itself. Moreover, while the exogenous expression of an RNAi-resistant full length MCPH1-transcript was sufficient to rescue this effect of the MCPH1 knockdown, a transcript that was isolated in a human breast cancer specimen lacking the C-terminal BRCT-domains was not able to complement the defect. It was also reported that microcephalin/BRIT1 is involved in transcriptional activation of many important genes of the DNA damage response. Interestingly, it was shown that the C-terminal BRCT domains are also essential in exerting this function.

Hence, it remains to be explained why our investigations show that repair foci formation, G2/M checkpoint control, and chromosomal breakage rates in the Mcph1^gt/gt^ cell cultures are not affected by the genetrap mutation deleting the C-terminal BRCT-domain. Also the expression profiling of the Mcph1^gt/gt^ mice did not show a shifted expression of key components of the DNA damage response network, such as BRCA1 and CHK1 that were published to be affected by MCPH1 knockdowns.

Certainly, again, it may be possible that residual wild type protein or even the truncated protein encoded by the gene trap allele exert these functions. However, also the depletion of wild type Mcph1 and Mcph1^gt^ by RNAi did not impair the formation of 53BP1 foci neither in wt nor in Mcph1^gt/gt^ cells. Although it may be possible that the knockdown in our experiments was not efficient enough to induce the damage response phenotype, this seems unlikely as it was efficient enough to induce defective chromosome condensation in wt cells.

Therefore, although it was shown that Mcph1 co-localizes with γ-H2AX in repair foci also in mice cells [17], we can not ultimately rule out that in some aspects the role of Mcph1 in DNA damage response may differ between mice and human. However, also the function of Mcph1 in the regulation of chromosome condensation may be more dosage sensitive than some of its other functions. We can also not exclude that the use of radiomimetics may result in different results compared to double strand break induction by ionizing irradiation.

In this context it seems noteworthy, that we did not observe radiosensitivity in human MCPH1 patient cell lines bearing homozygous truncating mutations (T143NfsX5) [4] and that 53BP1 also forms regular DNA repair foci that co-localize with γ-H2AX in MCPH1 patient fibroblasts (our own unpublished observations).

Further experiments will be necessary to explain these conflicting results. The more so as, while our manuscript was in revision, another report on the functions of MCPH1 in DNA damage response was published and the authors of this study equally did not observe an effect of the depletion of hMCPH1 on 53BP1 foci formation [36].

In conclusion, we describe here the first *Mcph1* mouse model. We could show that a mutation caused by a gene trap vector results in impaired Mcph1 function. Evidence comes from the confirmation of the defect in the regulation of chromosome condensation in proliferating cells of the Mcph1^gt/gt^-mice. This defect resembles that described for PCC syndrome a human genetic disorder caused by *MCPH1* mutations. Most importantly, the overall life span of the Mcph1^gt/gt^ is significantly shortened. Thus, the mouse model should prove useful for the further delineating of the consequences of *MCPH1* mutations on patient's health and to unravel the molecular mechanisms which determine microcephalin/BRIT functions.

## Supporting Information

Figure S1 Thymidine pulse labeling. Cell cycle analysis after 3H-thymidine-pulse-labeling of logarithmically growing transformed fibroblast cell lines from Mcph1^gt/gt^ (grey diamonds) and WT animals (white squares). One hour after the pulse the percentage of labeled prophases and/or prophase-like cells is significantly higher in the Mcph1^gt/gt^ cells compared to control cells.
(0.17 MB TIF)

Figure S2 p-H3-labeling. An exemplary normal prophase cell displaying full histone H3-(Ser10) phosphorylation.
(0.94 MB TIF)

Figure S3 Determination of the depletion success. Mcph1 and Mcph1^gt^ were depleted by RNAi. (A) The depletion induced the cellular phenotype of defective chromosome condensation in the wt cells. Quantitative analysis of the proportion of prophase-like cells to the left and exemplary prophase-like cells from the RNAi treated wt cell cultures to the right. (B) Relative quantification of the wild type Mcph1 allele following RNAi in wild type and Mcph1^gt/gt^ cells by quantitative real time PCR. (C) Relative quantification of the genetrap allele following RNAi in Mcph1^gt/gt^ cells by quantitative real time PCR.
(0.35 MB TIF)

Figure S4 Expression profiling of Mcph1^gt/gt^ tissues. (A) Venn-diagram of genes upregulated (P-value ≤0.05) in different tissues of Mcph1^gt/gt^ mice and (B) Venn-diagram of genes downregulated (P-value ≤0.05) in different tissues of Mcph1^gt/gt^ mice.
(0.18 MB TIF)

Table S1 Downregulated genes (Diff_Score ≤−13/P≤0.05) in different Mcph1^gt/gt^ tissues.
(0.33 MB XLS)

Table S2 Upregulated genes (Diff_Score ≥13/P≤0.05) in different Mcph1^gt/gt^ tissues.
(0.57 MB XLS)

Table S3 Expression of potential Mcph1-targets in brain of Mcph1^gt/gt^ mice.
(0.02 MB XLS)

Table S4 Expression of potential Mcph1-targets in liver of Mcph1^gt/gt^ mice.
(0.02 MB XLS)

Table S5 Expression of potential Mcph1-targets in spleen of Mcph1^gt/gt^ mice.
(0.02 MB XLS)

Table S6 Comparing protein expression changes between Mcph1^gt/gt^ and mouse models of three common neurodegenerative diseases.
(0.02 MB DOC)
