# Supplementary figures and images for: Establishment of a Mouse Model with Misregulated Chromosome Condensation due to Defective Mcph1 Function
Source: PLoS One. 2010 Feb 16;5(2):e9242. doi: 10.1371/journal.pone.0009242 (PMC2821930; doi:10.1371/journal.pone.0009242)

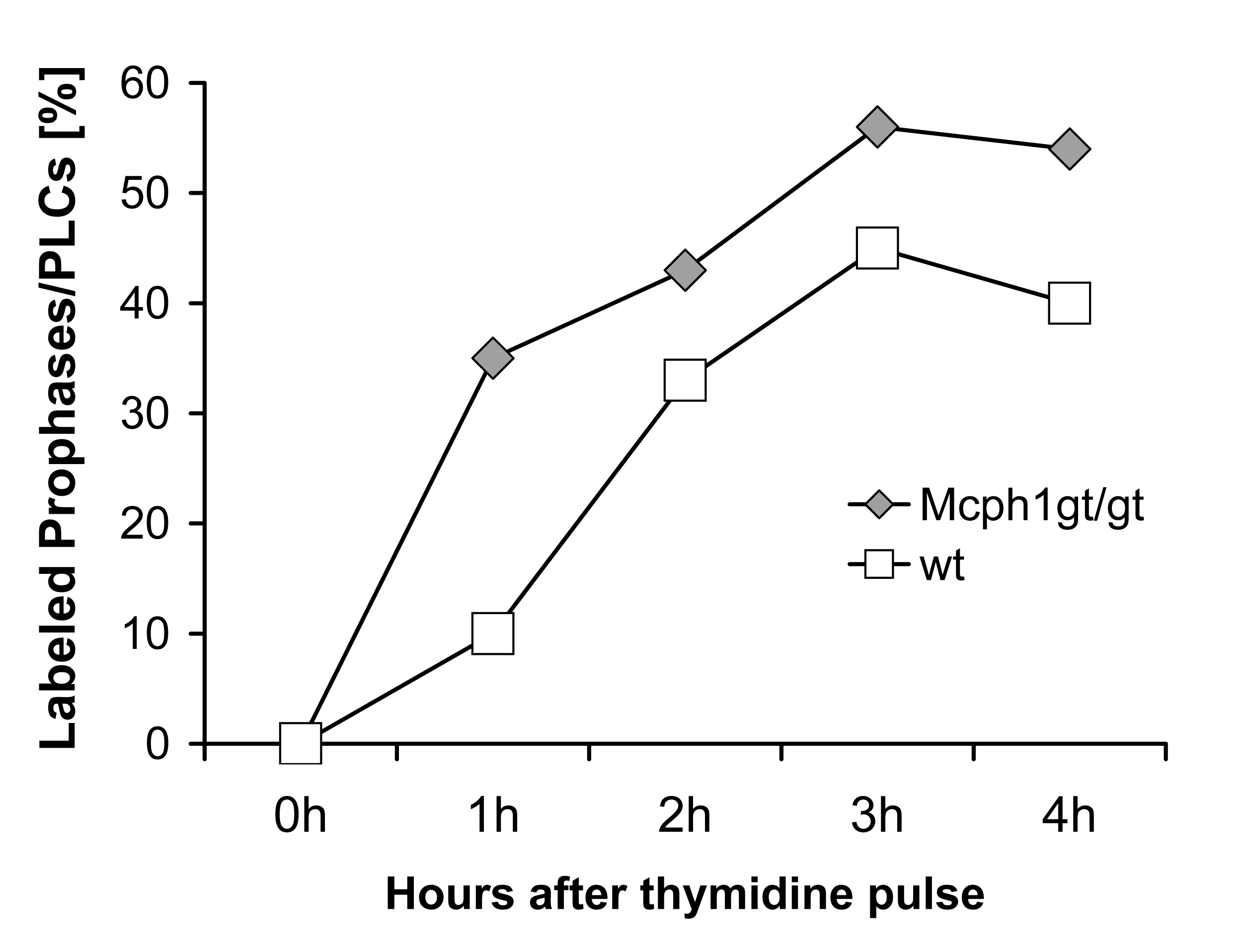

Supplement: Figure S1 — Thymidine pulse labeling. Cell cycle analysis after 3H-thymidine-pulse-labeling of logarithmically growing transformed fibroblast cell lines from Mcph1gt/gt (grey diamonds) and WT animals (white squares). One hour after the pulse the percentage of labeled prophases and/or prophase-like cells is significantly higher in the Mcph1gt/gt cells compared to control cells. (0.17 MB TIF) [file pone.0009242.s001.tif]

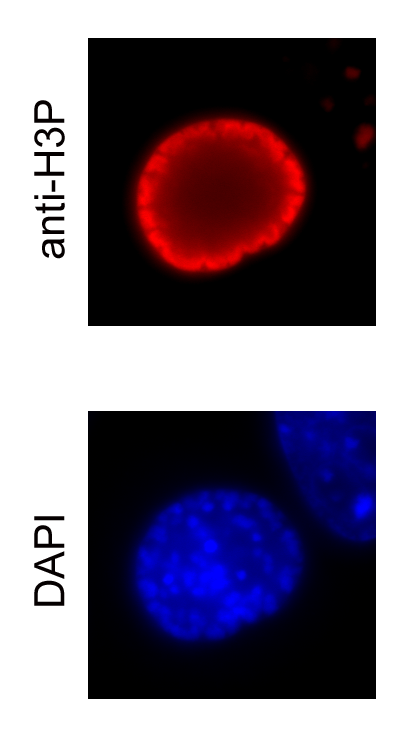

Supplement: Figure S2 — p-H3-labeling. An exemplary normal prophase cell displaying full histone H3-(Ser10) phosphorylation. (0.94 MB TIF) [file pone.0009242.s002.tif]

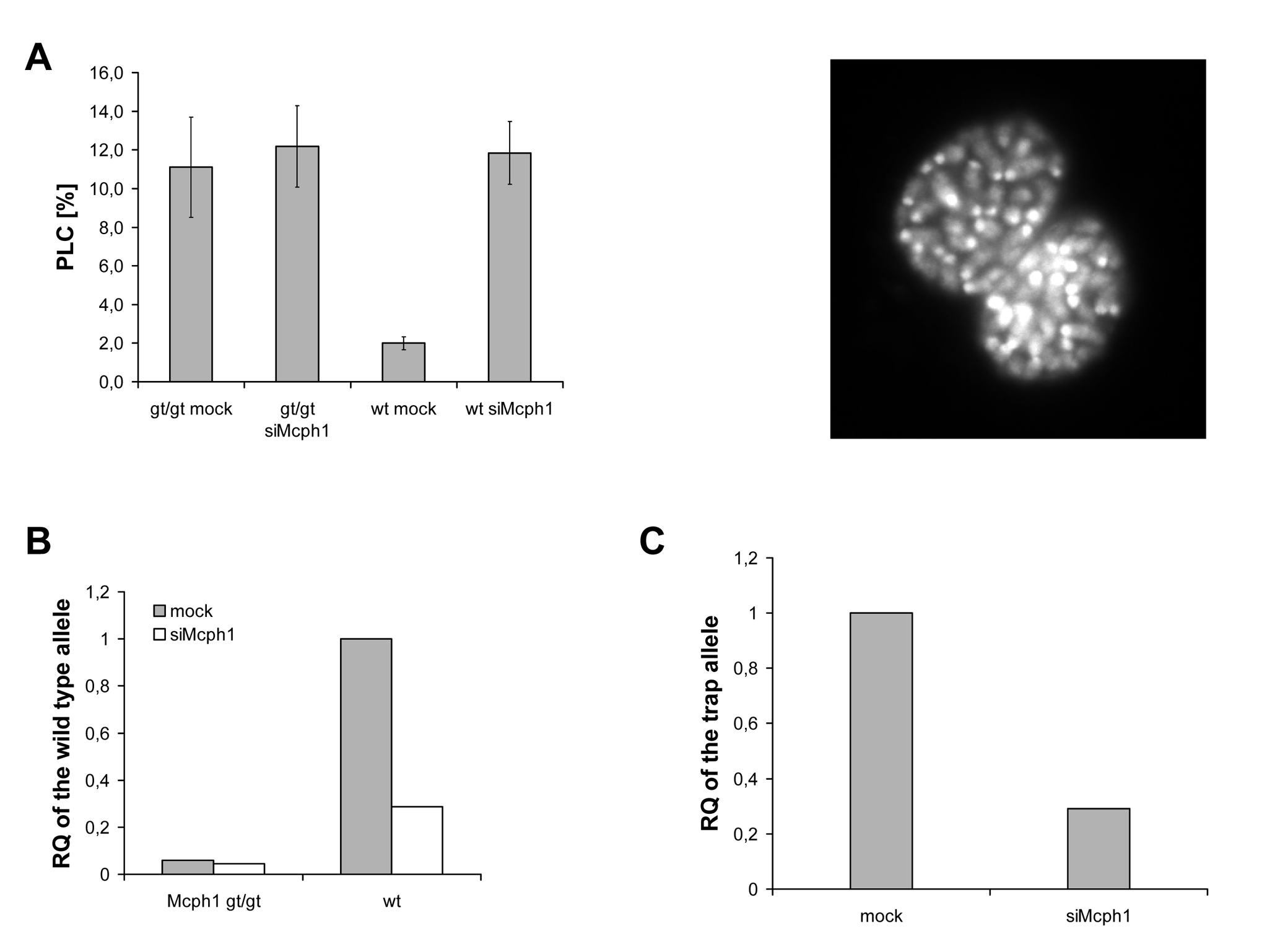

Supplement: Figure S3 — Determination of the depletion success. Mcph1 and Mcph1gt were depleted by RNAi. (A) The depletion induced the cellular phenotype of defective chromosome condensation in the wt cells. Quantitative analysis of the proportion of prophase-like cells to the left and exemplary prophase-like cells from the RNAi treated wt cell cultures to the right. (B) Relative quantification of the wild type Mcph1 allele following RNAi in wild type and Mcph1gt/gt cells by quantitative real time PCR. (C) Relative quantification of the genetrap allele following RNAi in Mcph1gt/gt cells by quantitative real time PCR. (0.35 MB TIF) [file pone.0009242.s003.tif]

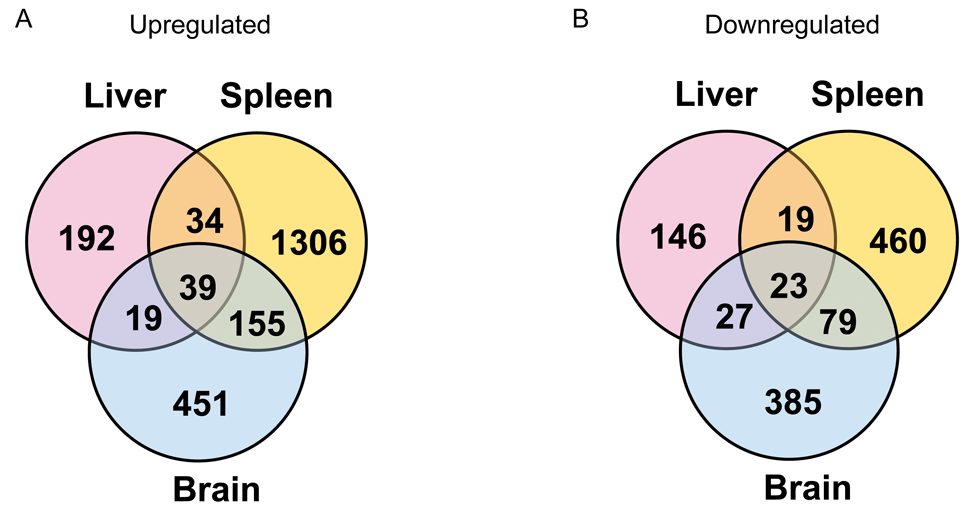

Supplement: Figure S4 — Expression profiling of Mcph1gt/gt tissues. (A) Venn-diagram of genes upregulated (P-value ≤0.05) in different tissues of Mcph1gt/gt mice and (B) Venn-diagram of genes downregulated (P-value ≤0.05) in different tissues of Mcph1gt/gt mice. (0.18 MB TIF) [file pone.0009242.s004.tif]
