# Supplementary material for: Establishment of a Mouse Model with Misregulated Chromosome Condensation due to Defective Mcph1 Function
Source: PLoS One. 2010 Feb 16;5(2):e9242. doi: 10.1371/journal.pone.0009242 (PMC2821930; doi:10.1371/journal.pone.0009242)
Supplement: Table S6 — Comparing protein expression changes between Mcph1gt/gt and mouse models of three common neurodegenerative diseases. (0.02 MB DOC) [file pone.0009242.s010.doc]

| Disease | brain region | Age | Upregulated | Downregulated | Total | Total spots | [%] Total spots |
| --- | --- | --- | --- | --- | --- | --- | --- |
| Mcph1^gt/gt^ | cortex | 15 weeks | 11 | 52 | 63 | 4006 | 1.6 |
| HD | total | 12 weeks | 120 | 120 | 240 | 4283 | 5.6 |
| AD | hippocampus | 15 months | 42 | 40 | 82 | 1769 | 4.6 |
| PD | midbrain | 10-15 weeks | 66 | 87 | 153 | 3293 | 4.6 |

HD = Huntington’s disease, AD = Alzheimer’s disease, PD = Parkinson’s disease
